# Challenges with medical education in Nigeria in the COVID-19 era

**DOI:** 10.11604/pamj.2020.37.223.26418

**Published:** 2020-11-06

**Authors:** Aishat Temitope Oladipo, Oluwayemisi Tolulope Fashola, Eniola Ifedolapo Agboola, Omolola Olayeni Adisa, Oluwatobiloba Dorcas Oyekanmi, Adeseye Micheal Akinsete

**Affiliations:** 1 Faculty of Clinical Sciences, College of Medicine, University of Lagos, Lagos, Nigeria,; 2 Department of Pediatrics, College of Medicine, University of Lagos/Lagos University Teaching Hospital, Lagos, Nigeria

**Keywords:** Coronavirus disease-2019 (COVID-19), medical education, Nigeria

## Abstract

On March 20^th^ 2020 the Federal Government of Nigeria ordered the closure of all educational institutions, this was inclusive of all medical schools in the country. During the initial phases of this closure, most institutions were at a loss on how to proceed with learning as universities in Nigeria use mainly the didactic lecturing model. As the lockdown progressed it became imperative to the institutions to set up e-learning media for continued instruction of students. It was found that in the institutions with e-learning facilities, the preclinical years remained mostly unaffected by the closure of medical schools due to the COVID-19 pandemic, while all institutions came to a standstill as regards providing a suitable alternative for clinical exposure. This therein has caused loss of valuable time and a change in the calendar of the school year, making it uncertain as to when the next set of qualified medical professionals will emerge in Nigeria. In this essay, we take a look at medical education in Nigeria, its challenges and progression in the COVID-19 era. We also take a look at the effect of the pandemic on learning and the subsequent interventions introduced to mitigate it.

## Essay

**Medical education in Nigeria:** medical education commenced in the University College Hospital (UCH) Ibadan in 1948 and has since then evolved over subsequent generations. Most of the newer medical schools adopted the curriculum of the older schools with little or no modification [1]. Several years after establishment, most of the medical schools have not revised their curricula unlike their counterparts in other regions of the world. Currently in Nigeria, there exist 42 medical schools of which; 17 are Federal, 18 are State institutions and 7 are privately owned. Globally, it has been reported that the most populated regions and countries have most of the medical schools; India, US, China, Brazil, Japan, Mexico, Pakistan and Russia account for over 40% of the total 2,409 recognized and operating medical schools in approximately 180 countries. The fastest growth rate was noticed in sub-Sahara Africa, the Caribbean, South Asia and South America [2]. Historically in Nigeria, the first three years of medical education consist of didactic lectures in the classrooms with the subsequent 3 years consisting of interactions within the confines of the hospital in the form of patient interactions and ward rounds. This format of medical education has held this way for the past half a century across most institutions in the country.

Thus, with the advent of the COVID-19 pandemic, there was no room for off-site or digital education in many of the medical training institutions in Nigeria. Adapting medical school in Nigeria to function efficiently during this pandemic has been an especially difficult endeavor because of the absence of infrastructure for digital learning. This commentary focuses on the need to review the curriculum across most medical training institutions in the country and introduce a dynamic digital component without diluting the core concept of the rigorous medical education. According to the Nigerian medical association, Nigeria produces 3,000 doctors annually with about 41,000 doctors currently working in the country. Data from the World Health Organization (WHO) has revealed that the doctor to patient ratio in Nigeria is four doctors per 10,000 patients, in comparison to other countries like the US and the UK with ratios of 26 and 28 doctors per 10,000 patients respectively [3]. Thus, it is important that Nigeria increases her capacity to produce doctors. Due to the COVID-19 pandemic, students have lost 7 months and counting as a result of the lockdown of services and institutions by the government. This may very well lead to inability to graduate doctors this year further worsening the doctor to patient ratio. The aim of this commentary is to highlight the challenges of face-to-face learning and proffer solutions that will accelerate the incorporation of digital learning platforms.

**Historical perspective of medical education in Nigeria:** during the pre-colonial era in Nigeria, medical care was essentially provided by traditional practitioners, who were trained as apprentices under relatives who were conversant with the art of healing. This is still the major source of medical care in some parts of Nigeria [4]. The first attempt at medical education in Nigeria was in 1927 when the government set up an institution to train medical manpower to diploma level [4]. This institution had a five-year training program like British medical schools; but it was ineffective as the teachers and facilities that were available were inadequate to train doctors acceptable outside the country. The ineffectiveness of this institution, resulted into its abolishment [4]. Formal medical education began with the establishment of UCH Ibadan, a college branch of the University of London in 1948 [1]. This institution was to train premedical students in Ibadan, preclinical students in Lagos with the clinical years at Ibadan [4]. The graduates were to be trained at the same level as the British and thereafter awarded a degree of the University of London. However, the general hospital that was to function as a teaching hospital was inadequate and could not meet the standard of the University of London. Thus, a decision was taken to establish an appropriate teaching hospital, the UCH Ibadan [4].

The very first set of medical students to graduate from Ibadan, Nigeria did so in the year 1960 utilizing the curriculum of the University of London with slight modifications in pediatrics with a focus on tropical diseases [4]. Medical education in Nigeria expanded between 1960-1972 as medical schools were established in Lagos, Zaria, Enugu, Benin and Ile-Ife [5]. The period from 1972-1998 saw further creation of additional medical schools at Ago-Iwoye, Ekpoma, Port Harcourt, Calabar, Nnewi, Ilorin, Jos, Kano, Sokoto, Maiduguri and Ogbomoso. These were regarded as the third generation of medical schools in Nigeria [4,5]. Each of these medical schools developed standards and curricula that were very similar to that of the first medical school at Ibadan with only slight adjustments. Medical education has since then expanded rapidly with the establishment of universities in almost every state in the country consisting of federal, state and private universities [5].

**Medical education during COVID-19 pandemic globally:** the onset of the COVID-19 pandemic ushered in the closure of universities and medical institutions globally. With the most effective strategy for the prevention of COVID-19 being social distancing in the absence of a vaccine, it is not certain how long this closure will last. This sustained closure of medical schools has created a challenge which e-learning platforms like WebEx (Cisco Webex, Milpitas, CA, USA) and Zoom may help to solve [6]. Medical education in many parts of the world is divided into pre-clinical and clinical years. With e-learning evolving has the mainstay during this period; it may be argued that pre-clinical medical education will not be significantly affected. The use of digital platforms for clinical engagements and electives may be difficult thus creating a significant gap in medical education. An innovative solution to this is the use of three-way telemedicine software which allow medical students to be a part of remote medical consultations [7]. This allows medical students take part in history taking, diagnosis and treatment of patients remotely. This will indirectly integrate telemedicine into the present medical curricula [7]. It will require dealing with issues around patient privacy and confidentiality. Other models which have been integrated in a bid to mitigate the effect of the pandemic to clinical medical education include case-based studies, problem-based learning in small virtual groups and use of simulators [7].

In a country like Turkey where medical education is transitioning into a more individualized model, physical presence is not required in the first 3 years except for laboratory sessions [8]. Virtual learning had been adopted so with the pandemic learning was not disrupted [8]. The transition was also easier in Hong Kong, with their previous history of suspension of clinical activities of medical students during SARS outbreak [9]. The model used then, which was highly accepted by their students involved the use of problem-based learning and case studies which has aided continuation of medical education in the course of this pandemic [9]. It is very impressive how innovative clinical academics have been in ensuring the continuity of medical education. However, all of these may not replace bed side teaching and authentic patient interactions. It is important to note that actual face to face interactions serve to inculcate skills such as communication, empathy, compassion as well as teamwork in medical students.

In resource constrained countries, the story is not the same with several challenges faced by medical educators in these settings hindering the continuity of medical education. In Brazil, it was observed that private universities were able to carry on by deploying electronic platforms which were not available to the public institutions. Unavailability of appropriate infrastructure and the presence of socially vulnerable students serve as a deterrence to online learning there [10]. The absence of sufficient IT infrastructure, uninterrupted internet access, stable power supply, access to computers by the socially vulnerable students and poor online content development by the faculty. The challenge of incorporating socially vulnerable students into the e-learning model in resource poor countries like Nigeria is a big one as it is a very costly process. There are problems such as high cost of internet subscription, unreliable internet service and epileptic power supply. This challenge brings forth certain questions: how do these schools incorporate virtual classrooms without exempting some students? Is e-learning feasible despite these infrastructural challenges? For final year medical students just short of graduation, will an extension of their medical training be warranted? Academics in these settings will have to make use of learning models that can bypass these problems to enable the provision of high-quality education to their medical students. What needs to be remembered is that the crisis of the global pandemic forced the transition into flipped classrooms not based on evidence on what works. Medical educators need to keep assessing this new form of educating medical students to determine if it could be continued after the pandemic abates.

**Medical education during COVID-19 pandemic in Nigeria:** on March 20^th^, 2020, the Federal Government of Nigeria ordered the closure of all educational institutions including tertiary institutions as a precaution to reduce the spread of COVID-19 in the country. In Nigeria, where medical education largely involves person to person interaction, this posed a great challenge to continuing medical education. To study the impact of this pandemic on medical education in Nigeria, a survey was carried out involving 72 medical students in different levels from medical schools throughout Nigeria. Medical students from public and private medical schools were included in a bid to get a full picture of the situation and also pick up on observable disparities in their medical education.

Findings from this survey showed that the majority (75%) of the participants stated that e-learning platforms were not used by their institutions prior to the pandemic. Interestingly, majority of those who stated that e-learning platforms were already in use were medical students from private institutions. With the mandatory closure of schools, 45% percent of the schools in this survey have managed to continue providing medical education to their students using various e-learning platforms. It was observed that all the private institutions continued education while only a few public institutions were able to continue providing education for their students. Furthermore, among the public medical schools, it was shown that the continuation of their education using e-learning platforms were available to some classes instead of all. The transition to e-learning by these medical schools was described to have taken about 4 - 8 weeks to put adequate structures in place. This shows that medical students were left with nothing to do for almost 2 months thereby creating a delay in their school calendar.

Exploring the e-learning model used by these various institutions, it was shown that multiple e-learning platforms were used including Zoom, WhatsApp, Google classroom, WebEx (Cisco WebEx, Milpitas, CA, USA) and institution owned platforms. Although the most commonly used platform was zoom, with a 75.6% use rate among the participants. The use of e-learning in medical education brings forth certain challenges when it comes to clinical clerkships and laboratory sessions. It was discovered that none of the schools had clear strategies to solve this problem as they deferred all clinical and laboratory sessions to when students that eventually return back to the clinical setting. In some schools, though clerkships had been deferred, a method of using pictures of disease conditions, videos of patient interactions and physical examinations were adopted for their medical students. The effectiveness of this method is uncertain.

Transition to the e-learning model was unprepared for in Nigeria but inevitable due to the pandemic. As a result of this unpreparedness and the situation of Nigeria as a resource limited country, this new method of learning comes with different challenges. It was interesting to note that the challenges highlighted by the students in private institutions largely differed from those in public institutions. Challenges noted by students in private institutions ranged from problems of poor internet connection to the inability of lecturers to use the digital platforms. While for students in public institutions, their challenges are two pronged, institutional and personal. Institutional challenges included; industrial strike of university lecturers, poor funding and administrative capacity, absence of existing e-learning platforms in schools, lack of technical support and knowledge, while the challenges influenced by the students include non-ownership of devices/gadgets to access virtual resources and online platforms by majority of the student population, high cost of internet service subscription and epileptic power supply.

Even though the private institutions appeared slightly more prepared for this transition, all the institutes are at a loss when it comes to graduating students without them completing their clinical exposure. Without the adequate infrastructure to bypass this challenge, the question is: will Nigeria induct final year students into the medical profession this year?

**Figure 1:**
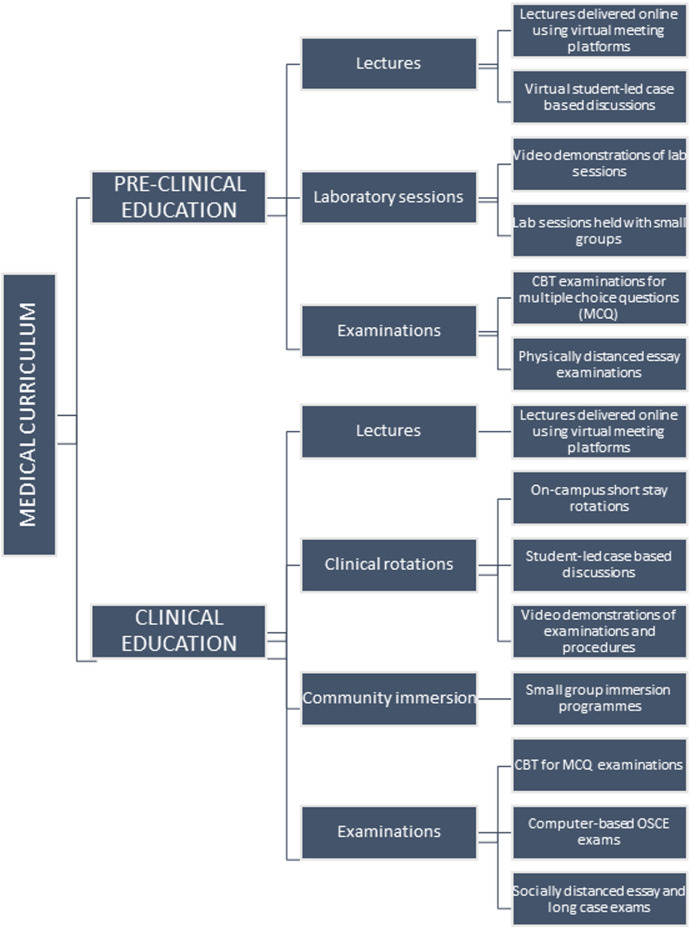
recommended modification of medical education curriculum in Nigeria

**Recommendations:** in light of the above, the following are recommended for the advancement of medical education in Nigeria (Figure 1): complete conversion of preclinical lectures to virtual learning through: online-based lectures using virtual meeting platforms; student led case-based discussions; video or live virtual demonstrations of laboratory sessions. Partial conversion of clinical lessons to virtual learning through: online lectures using virtual meeting platforms; video demonstrations of procedures and examinations. Short stay on-campus in-person sessions for: small group laboratory sessions; on-campus short-stay rotations for clinical exposure and lessons; small group community immersion carried out under strict supervision and monitoring. Migration of exams to computer-based tests (CBT) through: computer based test (CBT)- type examinations for in-course assessments and multiple-choice questions (MCQ) examinations: computer based objective structured clinical examinations (OSCE); socially distanced essay examinations with progressive transition to computer based tests through training of personnel and students.

## Conclusion

It is important to blend the current curricula with virtual learning models to enhance the learning experience of the students as well as strengthen the system for any eventualities in future. There is also the need for appropriate investment in personnel, equipment and infrastructure that will allow education to continue seamlessly regardless of environmental circumstances.
